# Monitoring the efficacy of dendritic cell vaccination by early detection of ^99m^Tc-HMPAO-labelled CD4^+^ T cells

**DOI:** 10.1002/eji.201344337

**Published:** 2014-03-19

**Authors:** Ehsan Sharif-Paghaleh, John Leech, Kavitha Sunassee, Niwa Ali, Pervinder Sagoo, Robert I Lechler, Lesley A Smyth, Giovanna Lombardi, Gregory E Mullen

**Affiliations:** 1 Medical Research Council (MRC) Centre for Transplantation, King's College London, King's Health Partners, Guy's HospitalLondon, United Kingdom; 2 Division of Imaging Sciences and Biomedical Engineering, King's College London, St. Thomas HospitalLondon, United Kingdom

**Keywords:** Adoptive transfer therapy, Non-invasive imaging, SPECT/CT, T-cell imaging, ^99m^Tc-HMPAO

DC vaccines have been used to induce tumour-specific cytotoxic T cells 1. However, this approach to cancer immunotherapy has had limited success. To be successful, injected DCs need to migrate to the LNs where they can stimulate effector T cells 1. We and others have previously demonstrated by MRI that tumour antigen-pulsed-DCs labelled ex vivo with superparamagnetic iron oxide nanoparticles migrated to the draining LNs and are capable of activating antigen-specific T cells 2, 3. The results from our study demonstrated that ex vivo superparamagnetic iron oxide nanoparticles-labelled and OVA-pulsed DCs prime cytotoxic CD8^+^ T-cell responses to protect against a B16-OVA tumour challenge. In the clinic, a possible noninvasive surrogate marker for efficacy of DC vaccination is to image the specific migration and accumulation of T cells following DC vaccination.

Mononuclear cells can be directly ex vivo radiolabelled with ^99m^Tc-Hexamethylpropyleneamine oxime (^99m^Tc-HMPAO) allowing the migratory pathway of adoptively transferred cells to be tracked by single photon emission computed tomography CT (SPECT/CT) 4. Here, we combined our previous experience with DC vaccination with the biodistribution in vivo of directly ^99m^Tc-HMPAO-labelled CD4^+^ T cells in response to OVA-pulsed DCs, using SPECT/CT imaging. This technology has its pitfalls, such as low cellular radiolabelling efficiency, but it has the advantage of being dramatically more sensitive than MRI thereby giving insight into early migration/accumulation of injected cells in vivo. For instance, a clinical study was recently stopped as the engineered melanoma-specific therapeutic T cells that were transferred into melanoma patients were cross reactive with an irrelevant antigen in the heart and caused death due to infiltration and proliferation in the heart 5. Imaging of the transferred T cells described above may have changed the outcome of the aforementioned study. Also, SPECT/CT can be used to monitor the function of DC vaccines by looking at T-cell migration and accumulation of injected T cells following DC vaccination. This is while this technology is non-invasive and possesses the ability to image deep in the tissue unlike intra-vital two-photon and bioluminescence imaging. In this study, we investigated the in vivo biodistribution of directly ^99m^Tc-HMPAO-labelled CD4^+^ T cells in response to OVA-pulsed DCs, as a model of tumour antigen, using SPECT/CT imaging.

T cells play an important role in protection against tumour invasion and T cells responses have been measured ex vivo following injection of tumour-antigen pulsed-DC vaccination 1. In order to determine if the efficacy of anti-cancer therapy can be assessed at early time points post-DC-vaccination in vivo, primary murine CD4^+^ T cells were radiolabelled ex vivo, injected and non-invasively imaged by SPET/CT. CD4^+^ T cells from DO11.10-Rag^−/−^ mice were isolated and radiolabelled with ^99m^Tc-HMPAO. The radiolabelling efficiency was between 1.1 and 8.5%. No difference in T-cell viability was observed between radiolabelled and non-radiolabelled cells (Supporting Information Fig. 1A). To determine the biodistribution of ^99m^Tc-HMPAO labelled CD4^+^ T cells in vivo in the absence of antigen, radiolabelled cells were adoptively transferred i.v. into BALB/c mice and imaged using NanoSPECT/CT. As illustrated (Fig.1A and B and Supporting Information Fig. 1B), ^99m^Tc-HMPAO labelled CD4^+^ T cells were observed in the spleen 1 hour post-injection. After scanning the mice were culled and organs were dissected for radioactive ex vivo biodistribution analysis. The biodistribution data confirmed the presence of injected radiolabelled CD4^+^ T cells within the spleen of recipient mice (standard uptake value (SUV) = 28.17 ± 4.21) (Fig.1C). Radiolabelled cells were also present in the lungs 1 hour post-injection (SUV = 7.45 ± 5.75). A control group of mice received ^99m^Tc-HMPAO tracer only and showed predominately clearance to the bladder with significantly less uptake in the spleen (SUV = 0.67 ± 0.07, *p* = 0.001) as compared with the uptake of radiolabelled T cells (Fig.1D, Supporting Information Fig. 1C and D).

**Figure 1:**
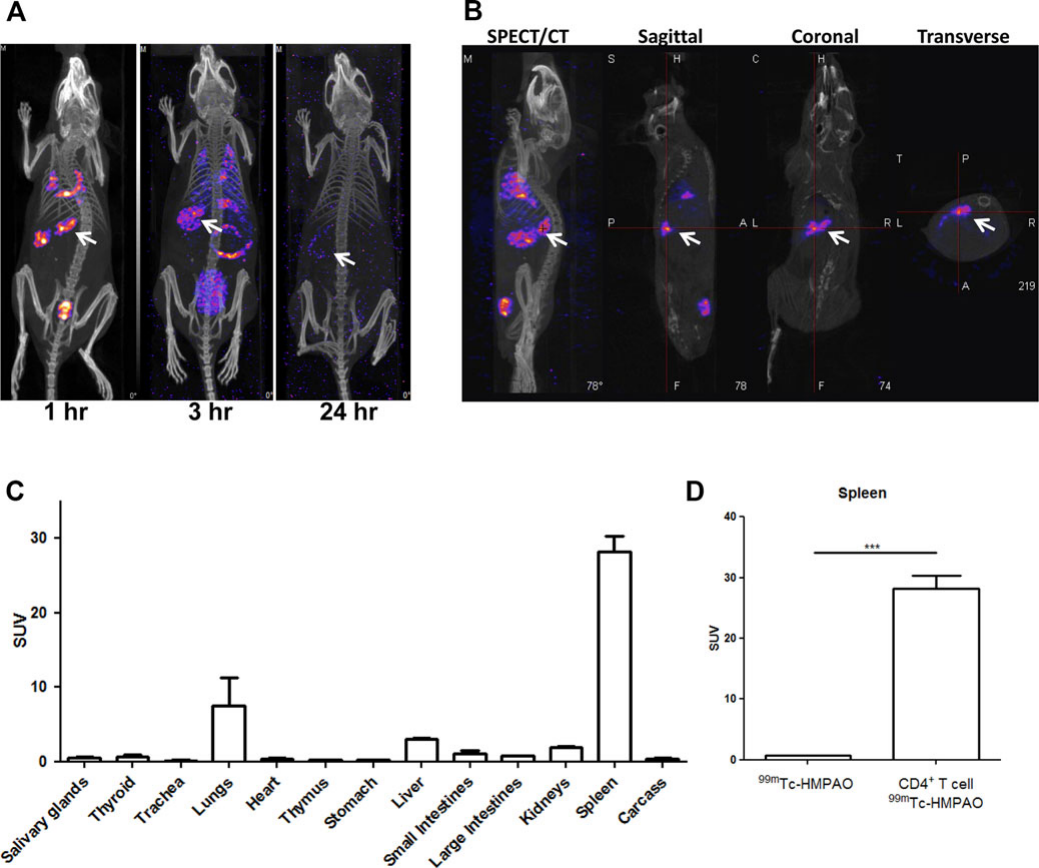
Whole body SPECT/CT imaging of directly ^99m^Tc-HMPAO radiolabelled CD4^+^ T cells. A total of 5 × 10^6^ freshly isolated CD4^+^ T lymphocytes from DO11.10-Rag^−/−^ mice were directly radiolabelled with ∼5 MBq of ^99m^Tc-HMPAO and adoptively transferred into BALB/c recipient mouse. (A) A representative SPECT/CT image of a mouse scanned at 1, 3, and 24 hours after adoptive transfer (*n* = 3 mice per time point). (B) SPECT/CT, sagittal, coronal, and transverse images of a mouse scanned after 1 hour after adoptive transfer. White arrows indicate the spleen. (C) After imaging, mice were culled and the biodistribution of ^99m^Tc-HMPAO CD4^+^ T cells were studied. Data are shown as mean + SEM of four mice pooled from three individual experiments performed. (D) The standard uptake value (SUV) of the spleens of mice receiving either ^99m^Tc-HMPAO or CD4^+^ T cells radiolabelled with ^99m^Tc-HMPAO was determined by measuring the presence of radioactivity in each organs and is shown as mean + SEM of four mice from three individual experiments. ***p = 0.0001, unpaired two-tailed *t* test.

Next, we investigated the migration and accumulation of T cells after DC vaccination. To achieve this, BALB/c mice were subcutaneously injected into the right and left lower legs with DCs pulsed or not with OVA peptide, respectively. After 24 hours, ∼10 MBq of ^99m^Tc-HMPAO and CFSE labelled DO11.10-Rag^−/−^ CD4^+^ T cells were intravenously injected (Fig.2A). SPECT/CT images were acquired from 0 to 3 hours post-injection and radiolabelled T cells were observed in the experimental right Inguinal LN (i.e., OVA pulsed DCs) but not in the control left Inguinal LN (non-pulsed DCs) (Fig.2B and Supporting Information Fig. 2A). To corroborate the in vivo data, the presence of T cells in the right LN was confirmed by ex vivo organ biodistribution and flow cytometry (Fig.2C and Supporting Information Fig. 2B). The analysis of the data demonstrated that OVA antigen-specific CD4^+^ T cells have migrated significantly more (*p* = 0.0008) to the site of antigen (right Inguinal LN) compared to the control site (left Inguinal LN). The specific recruitment of T cells to the OVA-bearing LN was further confirmed by flow cytometric analysis (Fig.2D), showing significantly more CFSE labelled CD4^+^ T cells in the right inguinal LN (1.01% ± 0.15) compared to the left inguinal LN (0.31% ± 0.14) (*p* = 0.0005). Altogether the results described here suggest that antigen-specific T cells can be radiolabeled with ^99m^Tc-HMPAO and subsequently imaged non-invasively at early time point using NanoSPECT/CT while remaining viable even with low labelling efficiencies.

**Figure 2:**
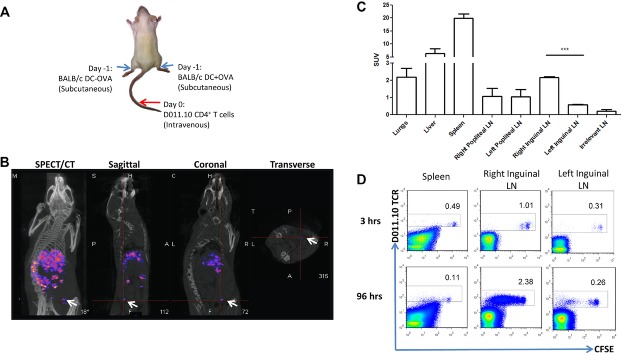
SPECT/CT imaging of antigen-specific T-cell response in vivo. (A) BALB/c-derived DCs were matured with LPS (1 μg/mL) and pulsed with 2 μg/mL of OVA peptide. The OVA-pulsed DCs were then subcutaneously injected (1 × 10^6^) into the right heel and unpulsed DCs were injected (1 × 10^6^) into the left heel. After 24 hours, 5 × 10^6 99m^Tc-HMAPO (∼10 MBq) radiolabelled CD4^+^ T cells isolated from DO11.10-Rag^−/−^ mice were intravenously injected. (B) After 3 hours the mice were imaged using NanoSPECT/CT. Radiolabelled CD4^+^ T cells migrated to the spleen as well as draining LN as indicated using white arrows (representative of three mice). (C) Mice were culled after scan and organs removed for biodistribution. The standard uptake value (SUV) in the indicated organs are shown as mean + SEM of four mice pooled from two individual experiments. ***p = 0.0008, unpaired two-tailed t test. (D) Flow cytometry analysis of cells present in the spleen, right and left inguinal LNs indicating the percentage of α-DO11.10 TCR and CFSE-labelled CD4^+^ T cells after 3 and 96 hours post-injection. Data shown are representative of four mice examined.

Direct ex vivo radiolabelling of leukocytes with radiotracers and gamma imaging is a routine clinical procedure within nuclear medicine 6. This has also previously been achieved in murine models by directly radiolabelling lymphocytes with ^111^In-oxine 7. Moreover, SPECT/CT was utilised to image the recruitment of HA-specific ^111^In-oxine-labelled CTL to HA expressing tumours 8. Also recently, de Vries et al. used ^111^In-Oxine labelled DCs pulsed with tumour antigen coupled with [^18^F]-labelled 3′-flouro-3′-deoxy-thymidine ([^18^F]FLT) PET imaging to detect antigen-specific immune responses against DC vaccine in melanoma patients 9. ^99m^Tc HMPAO has been used routinely for the direct radiolabelling of white blood cells for clinical imaging 4. However, it is not informative to compare the imaging of mixed population of cells (such as white blood cells) with that of enriched single population of cells. Interestingly, a recent study used ^99m^Tc HMPAO in imaging of pure eosinphils or neutrophils in humans 10. Here, we used the SPECT/CT technology to monitor the migration of T cells post-DC vaccination as a measure of the efficacy of DC vaccination.

We have shown in this study that direct radiolabelling of CD4^+^ T cells with ^99m^Tc-HMPAO did not induce significant cell death and that the radiolabelled T cells proliferate in vivo 4 days post-injection that is comparable to what we have previously reported 2. Although the radiolabelling efficiency was low, it was sufficient sensitive for visualising adoptively transferred radiolabelled CD4^+^ T cells in vivo using SPECT/CT. This was confirmed by organ biodistribution studies. This radiolabelling procedure and imaging via SPECT/CT could be a promising method of monitoring therapeutic intervention in man. Having shown previously that injected antigen-pulsed DCs migrated to draining LN 2, we used the same model to study T-cell activation and migration by adoptively transferred radiolabelled antigen-specific T cells one day after DCs vaccination. We demonstrate that migration of T cells to the draining LN was detected within 3 hours post-injection of 5 × 10^6^ cells. To our knowledge this is the first report of non-invasive imaging of early migration (i.e., within 3 hours post-injection) of CD4^+^ T cells to draining LN post-antigen challenge. In contrast, recruitment of CTLs to tumours in previously published murine models was imaged for example only after 24 hours post-injection of 10 × 10^6^ CTL 8. Also, in a clinical study where DCs were injected intranodally, ^111^In-labelled DCs were detected after 3 days and this was correlated with immune activation of T and B cells as these cells were activated and proliferated. Using ^18^F-FLT, the proliferation was detected using PET imaging. The direct radiolabelling method studied here can be beneficial when tracking of cells is required at early time points post-injection. However, direct radiolabelling of mononuclear cells using ^99m^Tc-HMPAO has its limitations with sometimes low and variable radiolabelling efficiencies as well as washout of the radiolabel from the cells 4. This can be overcome by indirect radiolabelling using a reporter genes, such as Sodium Iodide Symporter or Herpes Simplex Virus type 1 Thymidine Kinase 11. We have recently used the Sodium Iodide Symporter reporter gene to study the migration of murine Treg in mice and are currently correlating their capacity of inducing graft tolerance to their migratory property in a model of skin transplantation using SPECT/CT 12. Although this approach provides many advantages over direct radiolabelling methods, it requires gene modifications of cells and therefore is not broadly translated into the clinic.

## Abbreviations

SPECT/CT: single photon emission computed tomography CT
SUV: standard uptake value
^99m^Tc-HMPAO: ^99m^Tc-Hexamethylpropyleneamine oxime

## Supporting Information

The detailed *Materials and methods* for Technical comments are available online in the Supporting information

As a service to our authors and readers, this journal provides supporting information supplied by the authors. Such materials are peer reviewed and may be re-organized for online delivery, but are not copy-edited or typeset. Technical support issues arising from supporting information (other than missing files) should be addressed to the authors.

**Figure S1.** Whole body SPECT/CT imaging of ^99m^Tc-HMPAO radiolabelled CD4^+^ T cells. (A) Propidium Iodide stain on directly ^99m^Tc-HMPAO radiolabelled and un-labelled CD4^+^ T cells (representative of three experiments). (B) 5×10^6^ freshly isolated CD4^+^ T lymphocytes from DO11.10-Rag^−/−^ mice were directly radiolabelled with ∼5 MBq of ^99m^Tc-HMPAO and adoptively transferred into BALB/c recipient mouse. SPECT/CT movie of the kinetics of migration of adoptively transferred CD4^+^ T lymphocytes in recipient mice (representative of three mice per time point). (C) SPECT/CT movie of mice that were injected with 10 MBq of ^99m^Tc-HMPAO with no cells (representative of three mice). (D) *Ex vivo* biodistribution of mice that were injected with ^99m^Tc-HMPAO alone (n=4). **Figure. 2**. BALB/c derived DC were matured with LPS (1 μg/mL) and pulsed with 2 μg/mL of OVA peptide. The OVA pulsed DCs were then subcutaneously injected (1×10^6^) into the right heal and un-pulsed DCs were injected (1×10^6^) into the left heal. After 24hrs, 5×10^6.99m^ Tc-HMAPO (∼10 MBq) radiolabelled CD4^+^ T cells isolated from DO11.10-Rag^−/−^ mice were intravenously injected. After 3 hours the mice were imaged using NanoSPECT/CT. Radiolabelled CD4^+^ T cells migrated to the spleen as well as draining LN (representative of three mice). SPECT/CT imaging of the recruitment of antigen specific CD4^+^ T cells to the draining LN *in vivo* (representative of four mice) (A) and organ biodistribution analysis (from four mice) (B).

Peer review correspondence
