# Supplementary material for: Monitoring the efficacy of dendritic cell vaccination by early detection of 99mTc-HMPAO-labelled CD4+ T cells
Source: Eur J Immunol. 2014 Mar 19;44(7):2188–91. doi: 10.1002/eji.201344337 (PMC4211358; doi:10.1002/eji.201344337)
Supplement: Supplementary file 1 [file eji0044-2188-sd1.pdf]

European Journal of Immunology

Supporting Information

for

DOI 10.1002/eji.201344337

Ehsan Sharif-Paghaleh, John Leech, Kavitha Sunassee, Niwa Ali, Pervinder Sagoo, Robert I. Lechler, Lesley A. Smyth, Giovanna Lombardi and Gregory E. Mullen

Monitoring the efficacy of dendritic cell vaccination by early detection of ^{99m}Tc-HMPAO-labelled CD4^{+} T cells

© 2014 WILEY-VCH Verlag GmbH & Co. KGaA, Weinheim

SUPPORTING INFORMATION

SUPPORTING INFORMATION FIG. 1

Whole body SPECT/CT imaging of ^{99m}Tc-HMPAO radiolabelled CD4^{+} T cells. (A) Propidium Iodide stain on directly ^{99m}Tc-HMPAO radiolabelled and un-labelled CD4^{+} T cells (representative of three experiments). (B) 5x10^{6} freshly isolated CD4^{+} T lymphocytes from DO11.10-Rag$^{-/-}$ mice were directly radiolabelled with ~5 MBq of ^{99m}Tc-HMPAO and adoptively transferred into BALB/c recipient mouse. SPECT/CT movie of the kinetics of migration of adoptively transferred CD4^{+} T lymphocytes in recipient mice (representative of three mice per time point). (C) SPECT/CT movie of mice that were injected with 10 MBq of ^{99m}Tc-HMPAO with no cells (representative of three mice). (D) *Ex vivo* biodistribution of mice that were injected with ^{99m}Tc-HMPAO alone (n=4).

SUPPORTING INFORMATION FIG. 2

BALB/c derived DC were matured with LPS (1 µg/mL) and pulsed with 2 µg/mL of OVA peptide. The OVA pulsed DCs were then subcutaneously injected (1x10^{6}) into the right heal and un-pulsed DCs were injected (1x10^{6}) into the left heal. After 24hrs, 5x10^{6} ^{99m}Tc-HMAPO (~10 MBq) radiolabelled CD4^{+} T cells isolated from DO11.10-Rag$^{-/-}$ mice were intravenously injected. After 3 hours the mice were imaged using NanoSPECT/CT. Radiolabelled CD4^{+} T cells migrated to the spleen as well as draining LN (representative of three mice). SPECT/CT imaging of the recruitment of antigen specific CD4^{+} T cells to the draining LN *in vivo* (representative of four mice) (A) and organ biodistribution analysis (from four mice) (B).

Materials and methods

Mice, culture media, reagents, antibodies and Flow cytometric analysis

BALB/c (H-2^{d}) 6–8 weeks old mice were purchased from Harlan and DO11.10-Rag$^{-/-}$ mice were maintained under specific pathogen free conditions and all experiments were conducted in accordance with national guidelines for animal care. RPMI 1640 medium with L-glutamine, penicillin/streptomycin and 10% (v/v) FCS (Gibco, Invitrogen) were purchased from Invitrogen and anti-CD4–FITC (clone RM4-4) and anti-DO11.10 TCR-APC (clone KJ1-26) were from eBioscience. All flow cytometry analysis was conducted on a BD FACS LSR II (BD Biosciences). For surface staining, 5x10^{5} cells were incubated with saturating concentrations of

appropriate antibodies for 30 min at 4°C, washed twice in 4°C FACS buffer (PBS with 1% [v/v] FCS) before analysis.

Cell preparation

$CD4^+$ T cells: were generated from spleens of DO11.10-$Rag^{-/-}$ mice using Dynal CD4 negative isolation kit (Dynal, Invitrogen) according to the manufacturer's protocol. The purity of the $CD4^+$ T cells was assessed using flow cytometry and found to be greater than 94% for expression of CD4 (data not shown). $CD4^+$ T cells were then labelled with 1 μM of Carboxyfluorescein succinimidyl ester (CFSE, Molecular Probes, Invitrogen) and resuspended in saline solution before ^{99m}Tc-HMPAO radiolabelling.

DC: Bone marrow derived DCs (DCs) were generated according to the protocol described by Inaba and colleagues [11]. DCs were then activated with 1μg of Lipopolysaccharide (LPS, Alexis Biochemicals) on day 6 of culture. On day 7, half of the DCs were pulsed with 2 μg/mL of OVA peptide (Peptide International, peptide sequence ISQAVHAAHAEINEAGR) for 60 minutes at 37 °C before being washed and prepared for injection into recipient mice.

^{99m}Tc-HMPAO radiolabelling of $CD4^+$ T cells

500-600 MBq of $^{99m}TcO_4^-$ (kindly provided by Dept. of Nuclear Medicine, Guy's and St. Thomas' Hospital) reacted with HMPAO (Ceretec; GE Healthcare) was used to radiolabel $CD4^+$ T cells according to the manufacturer's protocol. The radiolabelling efficiency of the labelled cells was determined and resuspended in saline ready for injections. Varying radiolabelling efficiencies were obtained as mentioned in the manufacturer's protocol. Radiolabelled cells were also stained with Propidium Iodide (Molecular Probes, Invitrogen) to measure cell death using flow cytometry.

Adoptive transfer and nanoSPECT/CT imaging of $CD4^+$ T cells in vivo

BALB/c mice were injected intravenously (i.v) with $5x10^6$ DO11.10-$Rag^{-/-}$ $CD4^+$ T cells which were radiolabelled with ~5 MBq of ^{99m}Tc-HMPAO. The mice were then imaged after 1, 3 and 24 hr post injection, under inhaled isofluorane anaesthetics for 1 hr using a small animal SPECT/CT scanner (Manufactured by Mediso, Budapest, Hungary) equipped with a multipinhole (nine pinholes, aperture 1.0 mm) collimator. Images were then reconstructed using the VivoQuant software (inviCRO, Boston, USA). Mice were then culled and

^{99m}Tc-HMPAO *ex vivo* biodistribution study was performed. Standard uptake values of ^{99m}Tc-HMPAO were calculated by the formula: [CPM (organ)/weight (organ)] / [CPM (whole mouse)/weight (whole mouse)].

For the tracking of CD4$^+$ T cells to the draining LN experiment, BALB/c mice were subcutaneously injected in the right and left heal with 1x10^6 LPS-matured DC pulsed with or without OVA peptide. 24 hr later, 5x10^6 CFSE and ^{99m}Tc-HMPAO (~10 MBq) labelled DO11.10-Rag$^{-/-}$ CD4$^+$ T cells were adoptively transferred i.v. Three hours post-injection, mice were imaged for 60 min using NanoSPECT/CT as described above. After scans mice were culled and radioactive *ex vivo* biodistribution and flow cytometry studies were undertaken. Standard uptake values were calculated as described above. Some animals were culled 96 hours post i.v injection and the spleens and LN were analysed by flow cytometry.

Statistics

Data are expressed as mean ± Standard Error of the Mean (SEM). Significance between samples was calculated using unpaired 2-tailed t test using GraphPad Prism 5 software. P values less than 0.05 were considered statistically significant.

Acknowledgement:

The authors acknowledge the financial support from King's College London, Medical Research Council Centre of Transplantation, Division of Imaging Sciences, The Department of Health via the National Institute for Health Research (NIHR) comprehensive Biomedical Research Centre (BRC) award to Guy's and St Thomas' National Health Service (NHS) Foundation Trust in partnership with King's College London and King's College Hospital NHS Foundation Trust and The British Heart Foundation. We also thank Dr Raul Elgueta for reviewing this manuscript.

Ethical statement:

All the animal studies have been approved by the UK Home office legislation

The authors declare that they have no conflict of interest.
