# Supplementary material for: Monitoring the efficacy of dendritic cell vaccination by early detection of 99mTc-HMPAO-labelled CD4+ T cells
Source: Eur J Immunol. 2014 Mar 19;44(7):2188–91. doi: 10.1002/eji.201344337 (PMC4211358; doi:10.1002/eji.201344337)
Supplement: Supplementary file 2 — Peer review correspondence [file eji0044-2188-sd2.pdf]

European Journal of Immunology

Peer review correspondence

Monitoring the efficacy of dendritic cell vaccination by early detection of ^{99m}Tc-HMPAO-labelled CD4^{+} T cells

Ehsan Sharif-Paghaleh, John Leech, Kavitha Sunassee, Niwa Ali, Pervinder Sagoo, Robert I. Lechler, Lesley A. Smyth, Giovanna Lombardi, Gregory E. Mullen.

Corresponding author: Gregory E. Mullen, Medical Research Council (MRC) Centre for Transplantation, King's College London

---

Review Timeline:	Submission date:	26 November 2013
	First editorial decision:	6 January 2014
	First revision received:	11 January 2014
	Second editorial decision:	6 February 2014
	Second revision received:	12 February 2014
	Accepted:	12 March 2014

Handling Executive Committee member: Prof. Reinhold Förster

Please note that the correspondence below does not include the standard editorial instructions regarding preparation and submission of revised manuscripts, only the scientific revisions requested and addressed.

First Editorial Decision – 6 January 2014

Dear Dr. Sharif-Paghaleh,

My apologies for the slight delay in processing the review of your manuscript ID eji.201344337 entitled "Early in vivo detection of 99mTc-HMPAO labelled CD4+ T cells to monitor the efficacy of dendritic cells vaccination" which you submitted to the European Journal of Immunology due to the holiday season. Your technical comment has been reviewed and the comments of the referees are included at the bottom of this letter.

A revised version of your manuscript that takes into account the comments of the referees will be reconsidered for publication.

You should also pay close attention to the editorial comments included below.

Please note that submitting a revision of your manuscript does not guarantee eventual acceptance, and that your revision will be re-reviewed by the referees before a decision is rendered.

If the revision of the paper is expected to take more than three months, please inform the editorial office. Revisions taking longer than six months may be assessed by new referees to ensure the relevance and timeliness of the data.

Once again, thank you for submitting your manuscript to European Journal of Immunology and we look forward to receiving your revision.

Yours sincerely,
Karen Chu

On behalf of Prof. Reinhold Forster

Dr. Karen Chu
Editorial Office
European Journal of Immunology
e-mail: ejied@wiley.com

****

Reviewer: 1

Comments to the Author
In this manuscript, the authors analyze the redistribution behavior of adoptively transferred T cells, using whole body spect/ct analysis. The work presented here reveals beautiful full body overviews of the preferred locations, where CD4+ T cells home after transfer. The authors show that radioactive labeling of T cells maintains their viability and reveal in their first set of analyses that the preferred organs of homing are the spleen and lungs. While this information is not novel, the technique presented here is showing its power. The visual analysis is confirmed by classical radioactive measurements of isolated organs indicating the specificity and reliability of the approach.

This reviewer wonders about the correct choice of the experimental approach. Especially in figure 2 and supplemental figure 2, the authors injected antigen labeled dendritic cells in the lower right leg and antigen-free dendritic cells in the lower left leg. Later they analyse recruitment of CD4+ T cells to the inguinal lymph nodes. Wouldn't immunisation into the flank rather than the leg target dendritic cells to inguinal lymph nodes? This is only a minor critique, as the authors obtain a certain amount of T-cell

European Journal of Immunology

recruitment to the correct LN of the body. This reviewer just wonders if the results could be optimized if the correct lymph nodes (for example popliteal after footpath-, or inguinal after flank-immunisation) would have been chosen. Also for the injected Ag-labeled dendritic cells such a migratory pathway might be more obvious rather than migrating from the leg to the inguinal lymph node.
Another minor critique is the insufficient labeling of the figure legends for the supporting information fig 1 and 2. Without reading the results section it is not at all clear which experiment is described in supporting figure 2.

Reviewer: 2

Comments to the Author
The authors use a direct radiolabelling method already described by others (cited ref 4) for human PBMC for labelling of murine bulk CD4+ T cells which were adoptively transferred 24 h after injection of ova peptide labelled DC into the right and of unloaded DC into the left leg. As expected within a few hours T cells were recruited and retained in the right inguinal nodes but not the left ones. Only short term observation was possible.

This manuscript does not provide any technical advances or tricks as such are not required for this standardized method which is in wide use for diagnostic purposes in human disease (ref 4).

One major problem is that the usefulness of the labelling method is not compared to any other method such as Indium labelling or PET imaging both of which have been used already successfully in man to monitor DC vaccination. Importantly, PET imaging has been used for early identification of antigen-specific immune responses in vivo in humans, but the respective work is not cited (Aarntzen EH, Srinivas M, De Wilt JH, Jacobs JF, Lesterhuis WJ, Windhorst AD,
Troost EG, Bonenkamp JJ, van Rossum MM, Blokx WA, Mus RD, Boerman OC, Punt CJ,
Figdor CG, Oyen WJ, de Vries IJ. Early identification of antigen-specific immune
responses in vivo by [18F]-labeled 3'-fluoro-3'-deoxy-thymidine ([18F]FLT) PET
imaging. Proc Natl Acad Sci U S A. 2011 Nov 8;108(45):18396-9.).

The authors also do not at all experimentally address or even discuss the issue that the labelling used will probably block the division of DC stimulated lymphocytes which would make it really difficult to use this method to monitor the EFFICACY of DC vaccination early on. The authors should discuss and experimentally address the statement given in ref 4 "Labelling of mixed leucocytes causes radiation damage to the lymphocytes as a result of self-irradiation by low-energy Auger electrons. However, since the lymphocytes are unable to divide after labelling and are eliminated through apoptosis and

European Journal of Immunology

phagocytosis, the risk of lymphoid malignancies after administration of 99mTc-HMPAO-labelled mixed leucocytes is considered to be negligible."

First Revision – authors' response – 11 January 2014

Reviewer 1:

Comments to the Author

In this manuscript, the authors analyze the redistribution behavior of adoptively transferred T cells, using whole body spect/ct analysis. The work presented here reveals beautiful full body overviews of the preferred locations, where CD4+ T cells home after transfer. The authors show that radioactive labeling of T cells maintains their viability and reveal in their first set of analyses that the preferred organs of homing are the spleen and lungs. While this information is not novel, the technique presented here is showing its power. The visual analysis is confirmed by classical radioactive measurements of isolated organs indicating the specificity and reliability of the approach.

We thank the reviewer for their positive comments.

==

This reviewer wonders about the correct choice of the experimental approach. Especially in figure 2 and supplemental figure 2, the authors injected antigen labeled dendritic cells in the lower right leg and antigen-free dendritic cells in the lower left leg. Later they analyse recruitment of CD4+ T cells to the inguinal lymph nodes. Wouldn't immunisation into the flank rather than the leg target dendritic cells to inguinal lymph nodes? This is only a minor critique, as the authors obtain a certain amount of T-cell recruitment to the correct LN of the body. This reviewer just wonders if the results could be optimized if the correct lymph nodes (for example popliteal after footpath-, or inguinal after flank-immunisation) would have been chosen. Also for the injected Ag-labeled dendritic cells such a migratory pathway might be more obvious rather than migrating from the leg to the inguinal lymph node.

The authors have also observed that if DCs pulsed with antigen are injected via foot pad they migrate to popliteal (draining) LN. However, due to restrictions by the author's UK animal project license, foot pad injections are not allowed (due to the severity of the procedure), instead the DCs are injected subcutaneously into the heal of mice (above the ankle) which then drains to inguinal LN.

===================================

Another minor critique is the insufficient labeling of the figure legends for the supporting information fig 1 and 2. Without reading the results section it is not at all clear which experiment is described in supporting figure 2.

The authors have taken this comment into full consideration and have now modified accordingly.

Reviewer 2:

Comments to the Author

The authors use a direct radiolabelling method already described by others (cited ref 4) for human PBMC for labelling of murine bulk CD4+ T cells which were adoptively transferred 24 h after injection of ova peptide labelled DC into the right and of unloaded DC into the left leg. As expected within a few hours T cells were recruited and retained in the right inguinal nodes but not the left ones. Only short term observation was possible.

This manuscript does not provide any technical advances or tricks as such are not required for this standardized method which is in wide use for diagnostic purposes in human disease (ref 4).

The authors are well aware of the use of Tc-99m HMPAO for the direct labelling of white blood cells for clinical imaging. However it is not informative to compare the imaging of mixed population of cells (such as WBC) with that of enriched single population of cells. Indeed the authors themselves have recently demonstrated the use of Tc-99mHMPAO in imaging of pure eosinphils or neutrophils in humans (Lukawska et al JACI 2013). This paper demonstrates that the >75% of Tc-99m HMPAO is taken up by eosinophils suggesting that mononuclear cells could be underrepresented in the imaging of WBC. However, this is not the point of the paper. The reviewer states “As expected within a few hours…” – it is expected that antigen specific T-cell will migrate to site of antigen i.e. draining LN, but it has not been shown before that this occurs as early as 3 hour post IV injection. The point of this paper is to demonstrate to immunologist that it is possible to investigate the migration and biodistribution of T-cells non-invasively within 3 hours post injection. Why is this important? Because it allows us to quantify the initial recruitment of parental T-effector cells as proposed to the traditional ex vivo measurement of proliferation of cells isolated typically 3-4 days post injection of T-cells. To our knowledge this is the first report of non-invasive imaging of early migration (i.e. within 3 hours post injection) of CD4+ T-cells to draining LN post antigen challenge.

================================

One major problem is that the usefulness of the labelling method is not compared to any other method such as Indium labelling or PET imaging both of which have been used already successfully in man to monitor DC vaccination. Importantly, PET imaging has been used for early identification of antigen-specific immune responses in vivo in humans, but the respective work is not cited (Aarntzen EH, Srinivas M, De Wilt JH, Jacobs JF, Lesterhuis WJ, Windhorst AD,Troost EG, Bonenkamp JJ, van Rossum MM, Blokx WA, Mus RD, Boerman OC, Punt CJ, Figdor CG, Oyen WJ, de Vries IJ. Early identification of antigen-specific immune responses in vivo by [18F]-labeled 3'-fluoro-3'-deoxy-thymidine ([18F]FLT) PET imaging. Proc Natl Acad Sci U S A. 2011 Nov 8;108(45):18396-9.).

While the use of In-111 Oxine has been used previously for the tracking of CD4 T-cells the concern with such an approach is the radiochemical toxicity of the Indium Oxine itself (Block SS. Fungitoxicity of the 8-

quinolinols. J Agric Food Chem1955;3(3):229–34.), as well as the radiotoxicity of In-111 as a result of the Auger electrons. This coupled with the GE Healthcare's decision to discontinue In-111 Oxine in the UK leaves only Tc-99m HMPAO as the sole licensed commercial product for cell tracking.

In addition, in the discussion (page 5, side line indicators 6-15 and 30-34) the authors have compared and discussed the In-111 radiolabelled T-cell to our method of radiolabelling. Furthermore, the authors have now cited Aarntzen et al in the paper and have added a section in the discussion to cover PET imaging biomarkers . Although it should be noted that to date there are no licensed commercial PET direct labelling agents.

======================================

The authors also do not at all experimentally address or even discuss the issue that the labelling used will probably block the division of DC stimulated lymphocytes which would make it really difficult to use this method to monitor the EFFICACY of DC vaccination early on. The authors should discuss and experimentally address the statement given in ref 4 "Labelling of mixed leucocytes causes radiation damage to the lymphocytes as a result of self-irradiation by low-energy Auger electrons. However, since the lymphocytes are unable to divide after labelling and are eliminated through apoptosis and phagocytosis, the risk of lymphoid malignancies after administration of 99mTc-HMPAO-labelled mixed leucocytes is considered to be negligible."

Given the very poor radiolabelling efficiencies of the murine CD4 cells (i.e. less than 10%) and hence low specific activity (i.e. Bq/cell) we know these levels of Tc-99m have no effect on T-cell proliferation. In this manuscript (Supporting Information FIG. 1A) we present data to demonstrate that there was no difference in viability between labelled and unlabelled cells. We also shown in Figure 2D that the radiolabelled T-cells continue proliferate in vivo 4 days post injection and that this is comparable to what we have previously reported (Tavare et al PLoS One 2011) Indeed we have also previously shown (Sharif-Paghaleh et al, PLoS One 2011) that murine CD4 cells are NOT affected by Tc-99m at far higher specific activities as used in here. In this manuscript we used less than 0.3 Bq/cell while in Sharif-Paghaleh et al, PLoS One 2011 we used 3 Bq/cell i.e. more than 10 fold higher. We showed that no difference compared to unlabelled cells was observed in phenotype, MTT, necrosis and function (proliferation and suppression). We did observe a significant drop in viability in Sharif-Paghaleh et al, PLoS One 2011 when 5-10 Bq/cell was used to label CD4 T-cells.

<u>Second Editorial Decision – 6 February 2014</u>

Dear Dr. Sharif-Paghaleh,

Thank you for submitting your revised manuscript ID eji.201344337.R1 entitled "Early in vivo detection of 99mTc-HMPAO labelled CD4+ T cells to monitor the efficacy of dendritic cells vaccination" to the

Peer review correspondence

European Journal of Immunology. Your manuscript has been re-reviewed and the comments of the referees are included at the bottom of this letter.

Although the referees have recommended publication, some revisions to your manuscript have been requested. Therefore, I invite you to respond to the comments of referee 2 and revise your manuscript accordingly.

You should also pay close attention to the editorial comments included below. *In particular, please edit your figure legends to follow Journal standards as outlined in the editorial comments. Failure to do this will result in delays in the re-review process.*

If the revision of the paper is expected to take more than three months, please inform the editorial office. Revisions taking longer than six months may be assessed by new referees to ensure the relevance and timeliness of the data.

Once again, thank you for submitting your manuscript to European Journal of Immunology. We look forward to receiving your revision.

Yours sincerely,
Karen Chu

on behalf of Dr. Reinhold Forster

Dr. Karen Chu
Editorial Office
European Journal of Immunology
e-mail: ejied@wiley.com

****

Reviewer: 1

Comments to the Author
No comments

Reviewer: 2

Comments to the Author

The authors have answered my questions well, and I must say that there response is what I missed AND STILL MISS in the paper as part of the discussion and arguments.

It is difficult to understand why the authors do not MAKE it proactively clear to those interested in translation that the report is an IMPORTANT technical advance and note not just a note one showing nice pictures.

I do not have time to check but if what the authors state is true it should be one argument strongly stated in the manuscript: " To our knowledge this is the first report of non-invasive imaging of early migration (i.e. within 3 hours post injection) of CD4+ T-cells to draining LN
post antigen challenge.

Furthermore, I think it would be important to emphasize that "We also shown in Figure 2D that the radiolabelled T-cells continue proliferate in vivo 4 days post injection and that this is comparable to what we have previously reported (Tavare et al PLoS One 2011)" so that this approach can be translated into clinical studies as the concerns inherent to other labels are invalid (after all in man one would not like to let antigen-laden DC find and kill antigen-specific T cells!).

Finally, the authors should emphasize that one cannot simply conclude from the PBMC labeling studies performed by others that labeling also works for enriched T cells by using their arguments put forward in the answer to reviewer 2: " However it is not informative to compare the imaging of
mixed population of cells (such as WBC) with that of enriched single population of cells. Indeed the authors themselves have recently demonstrated the use of Tc-99mHMPAO in imaging of pure eosinphils or neutrophils in humans (Lukawska et al JACI 2013).

The authors would have further strengthened their manuscript by providing preliminary data demonstrating that human T cells can be successfully labeled, but this remains the author´s choice.

<u>Second Revision – authors’ response – 12 February 2014</u>

Reviewer 2:

Comments to the Author

The authors have answered my questions well, and I must say that there response is what I missed AND STILL MISS in the paper as part of the discussion and arguments.

It is difficult to understand why the authors do not MAKE it proactively clear to those interested in translation that the report is an IMPORTANT technical advance and note not just a note one showing nice pictures.

I do not have time to check but if what the authors state is true it should be one argument strongly stated in the manuscript: " To our knowledge this is the first report of non-invasive imaging of early migration (i.e. within 3 hours post injection) of CD4+ T-cells to draining LN post antigen challenge.
The authors have taken this comment into consideration and have added the requested statement to the manuscript. Please see tracked change manuscript.

Furthermore, I think it would be important to emphasize that "We also shown in Figure 2D that the radiolabelled T-cells continue proliferate in vivo 4 days post injection and that this is comparable to what we have previously reported (Tavare et al PLoS One 2011)" so that this approach can be translated into clinical studies as the concerns inherent to other labels are invalid (after all in man one would not like to let antigen-laden DC find and kill antigen-specific T cells!).
The authors have taken this comment into consideration and have added the requested statement to the manuscript. Please see tracked change manuscript (page 5).
Finally, the authors should emphasize that one cannot simply conclude from the PBMC labeling studies performed by others that labeling also works for enriched T cells by using their arguments put forward in the answer to reviewer 2: " However it is not informative to compare the imaging of mixed population of cells (such as WBC) with that of enriched single population of cells. Indeed the authors themselves have recently demonstrated the use of Tc-99mHMPAO in imaging of pure eosinphils or neutrophils in humans (Lukawska et al JACI 2013).
The authors have taken this comment into consideration and have added the requested statement to the manuscript. Please see tracked change manuscript (page 5).
The authors would have further strengthened their manuscript by providing preliminary data demonstrating that human T cells can be successfully labeled, but this remains the author´s choice.
Although demonstrating that human T cells can be successfully labelled has not been addressed in this manuscript, the data presented represents novel information for the future preclinical and clinical labelling of CD4 T-cells. The authors also feel that although to undertake human work is absolutely essential this is beyond the scope of this paper.

Third Editorial Decision – 3 March 2014

Dear Dr. Sharif-Paghaleh,

It is a pleasure to provisionally accept your manuscript entitled "Monitoring the efficacy of dendritic cell vaccination by early detection of 99mTc-HMPAO-labelled CD4+ T cells" for publication in the European Journal of Immunology. For final acceptance, please follow the instructions below and return the requested items as soon as possible as we cannot process your manuscript further until all items listed below are dealt with.

European Journal of Immunology

Please note that EJI articles are now published online a few days after final acceptance (see Accepted Articles: http://onlinelibrary.wiley.com/journal/10.1002/(ISSN)1521-4141/accepted). The files used for the Accepted Articles are the final files and information supplied by you in Manuscript Central. You should therefore check that all the information (including author names) is correct as changes will NOT be permitted until the proofs stage.

We look forward to hearing from you and thank you for submitting your manuscript to the European Journal of Immunology.

Yours sincerely,
Karen Chu

on behalf of Dr. Reinhold Forster

Dr. Karen Chu
Editorial Office
European Journal of Immunology
e-mail: ejied@wiley.com
www.eji-journal.eu
